# A DNA Sequence Element That Advances Replication Origin Activation Time in *Saccharomyces cerevisiae*

**DOI:** 10.1534/g3.113.008250

**Published:** 2013-11-01

**Authors:** Thomas J. Pohl, Katherine Kolor, Walton L. Fangman, Bonita J. Brewer, M. K. Raghuraman

**Affiliations:** * Molecular and Cellular Biology Program, University of Washington, Seattle, Washington 98195; † Department of Genome Sciences, University of Washington, Seattle, Washington 98195; ‡ Office of Public Health Genomics, Centers for Disease Control and Prevention, Atlanta, Georgia 30329

**Keywords:** DNA replication, origins of replication, *Saccharomyces cerevisiae*, ARS, temporal regulation

## Abstract

Eukaryotic origins of DNA replication undergo activation at various times in S-phase, allowing the genome to be duplicated in a temporally staggered fashion. In the budding yeast *Saccharomyces cerevisiae*, the activation times of individual origins are not intrinsic to those origins but are instead governed by surrounding sequences. Currently, there are two examples of DNA sequences that are known to advance origin activation time, centromeres and forkhead transcription factor binding sites. By combining deletion and linker scanning mutational analysis with two-dimensional gel electrophoresis to measure fork direction in the context of a two-origin plasmid, we have identified and characterized a 19- to 23-bp and a larger 584-bp DNA sequence that are capable of advancing origin activation time.

The replication of eukaryotic chromosomes is temporally regulated, with different sections along a chromosome replicating, on average, at different times during S-phase. In the baker’s yeast, *Saccharomyces cerevisiae*, the times at which origins undergo activation, or fire, have been shown to contribute to this temporal regulation (Raghuraman *et al.* 2001; Yabuki *et al.* 2002; McCune *et al.* 2008). Two classes of origins have been described whose activation is delayed until late in S-phase; in both cases, the origins fire late because of their location in the genome. One class of late origins includes those located near telomeres, with *ARS501* near the right end of chromosome V being the prototype of this class (Ferguson *et al.* 1991, 1992). Late firing is not an intrinsic property of the origin: transfer to a circular plasmid results in activation in early S-phase. Moreover, when normally early-activated origins are inserted near a telomere, they become late activated (Ferguson and Fangman 1992; Wellinger *et al.* 1993). The replication timing of telomere proximal origins has been shown to be governed by the Ku complex (a heterodimer consisting of Yku70 and Yku80 subunits), the silencing chromatin protein Sir3 (Stevenson and Gottschling 1999), and the TG_1-3_ repeat binding protein Rif1 (Cosgrove *et al.* 2002; Lian *et al.* 2011). A second class of late origins is exemplified by the chromosome XIV origin, *ARS1412* (Friedman *et al.* 1996). Here, delayed activation of the origin is caused by unspecified nontelomere sequence elements in flanking DNA. These delay elements can influence the activation times of origins that are located up to 6 kb away.

These observations suggested that in the absence of external influences, origins are inherently early activated, and that later firing origins are delayed in their activation because of their particular surrounding sequences. However, it has recently been shown that centromeres function in *cis* to confer early activation on origins residing up to 20 kb away (Pohl *et al.* 2012; Natsume *et al.* 2013). In addition, some origins that are not close to centromeres owe their early activation time, at least in part, to the binding of transcription factors Fkh1 and Fkh2 up to 500 bp from the origins (Knott *et al.* 2012). These data indicate that early origin activation is not merely a default state but that DNA elements such as centromeres and forkhead protein binding sites found in flanking sequences can promote early origin activation through the binding of kinetochores and forkhead proteins, respectively. In this manuscript, we provide evidence for a third DNA sequence element capable of advancing the time of activation of nearby origins.

The DNA sequence capable of imparting early origin activation was found through analysis of a plasmid, pN&S, that contains two copies of *ARS1*, located 180° from each other and designated *ARS1^N^* and *ARS1^S^* [*ARS1 north* and *ARS1 south*, respectively (Brewer and Fangman 1994); Figure 1A]. In most cell cycles, only one of the two ARSs on the molecule is activated, and although the two copies of *ARS1* on pN&S are identical in sequence, they are activated differentially such that initiation at *ARS1^S^* is favored approximately 4:1 over initiation at *ARS1^N^*. The element causing this origin bias on plasmid pN&S has been localized by deletion analysis to the 3′ end of the *URA3* gene, which is near *ARS1^S^* (Figure 1A; Brewer and Fangman 1994). We have concentrated on two hypotheses to explain the bias in origin use, origin competence and origin timing.

**Figure 1:**
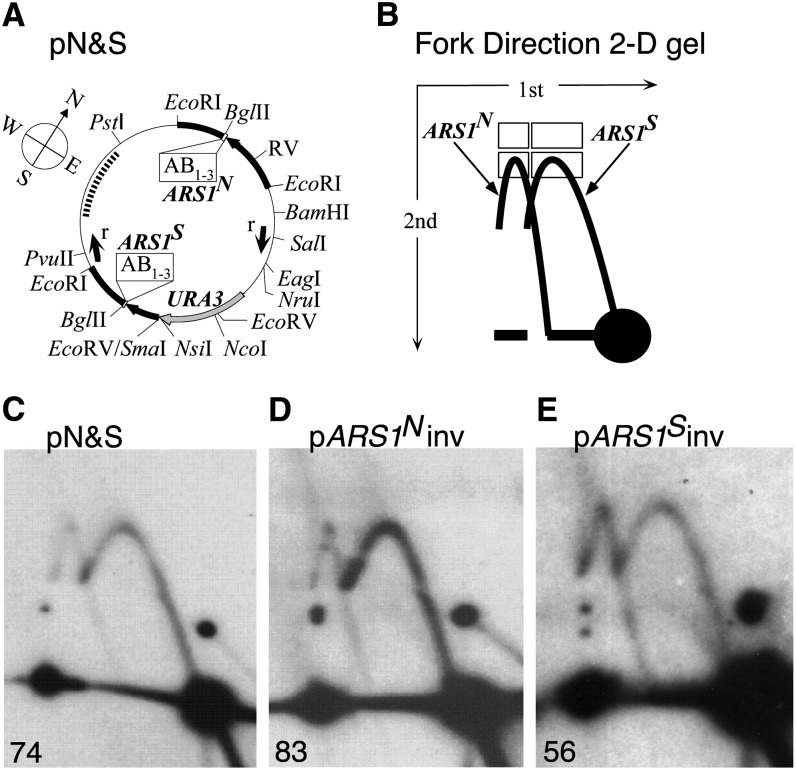
Fork direction analysis of the two-*ARS1* plasmid by 2-D gel electrophoresis. (A) Map of pN&S, a ~7.5-kb plasmid described previously (Brewer and Fangman 1994). Thick lines indicate sequences derived from yeast, whereas thin lines indicate prokaryotic vector sequences. The “compass” bearings (see text) are indicated by N, S, E, and W. A *Bgl*II site occurs immediately 3′ to the T-rich strand of the 11-bp ARS consensus sequence of each *ARS1*. The orientation of each copy of *ARS1* is indicated by the boxes containing letters representing the A and B regions (B_1-3_) defined for *ARS1* (Marahrens and Stillman 1992). The *URA3* gene is drawn as a gray arrow. The black arrows represent *TRP1* sequences. The *TRP1* gene fragment nearest to *ARS1^s^* lacks its promoter and 5′ sequences. The small arrows (labeled “r”) located in the plasmid’s interior represent the positions of the recombinase sites (cloned at the *Sal*I and *Pvu*II sites indicated) to make pN&Sdir (Figure S1). Not all sites for each enzyme are shown. The dotted line represents the region and the probe used to analyze fork direction 2-D gels. (B) Illustration depicting 2-D gel separation of forks moving in opposite directions through the southwestern *Bgl*II-*Pst*I fragment of pN&S. *Bgl*II digestion was carried out before electrophoresis in the first dimension and an in-gel digestion with *Pst*I before the second dimension. Forks emanating from *ARS1^N^* (left arc) are distinguished from the forks emanating from *ARS1^S^* (right arc). The efficiency of each ARS is expressed as a percentage of the total hybridization signal from an arc (two lower rectangles) after subtracting background hybridization (two upper rectangles). (C-E) Fork direction gels for (C) pN&S, (D) pARS1^N^inv (pN&S with the 1.45-kb *Eco*RI fragment containing *ARS1^N^* reversed), and (E) pARS1^S^inv (pN&S with the 1.05-kb *Eco*RI-*Eco*RV fragment containing *ARS1^S^* reversed). The numbers in the lower left of each panel refer to the percent initiation at *ARS1^S^* (see text for details).

The origin competence hypothesis is that the inherent biochemical ability of one of the *ARS1* copies to fire is affected because either its licensing or some subsequent initiation step is influenced by the 3′ *URA3* sequence. Consequently, either the ability of *ARS1^N^* to fire is reduced or that of *ARS1^S^* is increased. If this hypothesis were true, then the proposed difference in competence might be manifested as a difference in the ability of cells to maintain plasmids containing only the *ARS1^N^* region or the *ARS1^S^* region in the same context relative to the 3′ *URA3* sequences as plasmid pN&S. However, the observed relative rates of plasmid loss for such constructs do not reflect the initiation bias. A plasmid bearing *ARS1^S^*, which is predominantly used on pN&S, exhibits a slightly greater rate of loss than does a plasmid with *ARS1^N^* (Brewer and Fangman 1994).

An alternative hypothesis is that the 3′ *URA3* sequence causes *ARS1^S^* to fire earlier on average than *ARS1^N^*, resulting in passive replication of *ARS1^N^* before it has the opportunity to fire. A difference in the average time of activation of as little as one minute would be adequate to account for the passive replication of *ARS1^N^* in a majority of cells given that the distance between the two origins on plasmid pN&S is only ~3.75 kb and the average replication fork rate at 23° is about 3.5 kb/min (Rivin and Fangman 1980). This hypothesis more readily explains the absence of molecules in which both origins are active and is also consistent with the similar maintenance observed for plasmids containing either just the *ARS1^N^* region or just the *ARS1^S^* region.

In this study, we have dissected the bias element by deletion analysis and by scanning mutagenesis, and show that this element does indeed cause earlier activation in S-phase of the nearby copy of *ARS1* relative to the second, more distant copy of *ARS1*. Our results provide the identification of a potential third mechanism by which DNA sequences can advance origin activation time.

## Materials and Methods

### Strains and plasmids

Strain 3xRZeoDIR, which is RM14-3a (background A364a, *MATa*, *cdc7-1*, *bar1*, *his6*, *leu2-3,112*, *trp1-289*, *ura3-52*; McCarroll and Fangman 1988) with three copies of the 6891 bp *Cla*I fragment of pRINT (Friedman *et al.* 1995) tandemly integrated at the *LEU2* locus and a zeocin ARS-less cassette on the right arm of chromosome V, was used for the comparative hybridization experiments (Friedman *et al.* 1995). The ARS-less cassette was created by integrating the 3083 bp *Pvu*II fragment of pZeodir (described in the next paragraph) by homologous recombination. pN&S deletion derivatives were analyzed in RM14-3a or MR14-3a. Because the *trp1-1* allele of RM14-3a is somewhat revertible, MR14-3a, which is RM14-3a with a *TRP1* knockout allele, was used for all deletion constructs except pΔ43 and pΔ5. MR14-3a was used for all of the oligonucleotide mutagenesis constructs.

Plasmids were constructed in *Escherichia coli* strain DH5α. All bias-determinant plasmid constructs are based on plasmid pN&S (Brewer and Fangman 1994). Because of the duplication of the *TRP1ARS1* sequences in the two-*ARS1* plasmid, it was often difficult to find appropriate unique restriction sites for plasmid modifications. To circumvent this problem, several plasmids were constructed by a multistep process beginning with plasmids that contain only the south half of the plasmid. These plasmids are pBB3, pBB6, pBB6mut, and pBB6TA. pBB3 has the 967-bp *Nde*I-*Sma*I *URA3* fragment from YIp5 cloned into the 245-bp *Nde*I-*Sma*I fragment of pUC18. pBB6 has the 1068-bp *Eco*RI-*Eco*RV fragment of *TRP1ARS1* fragment cloned into the 3386-bp *Eco*RI-*Sma*I fragment of pBB3. pBB6TA has a 1453-bp *Eco*RI *TRP1ARS1* fragment cloned into the *Eco*RI site of pBB3 in the same orientation as pBB6. pBB6mut is pBB6TA with a 293-bp *Nde*I-*Nco*I deletion. After the desired alteration had been made, the two-ARS plasmid was reconstructed. pZeodir was constructed by ligating the 1181-bp blunt *Kpn*I-*Hin*dIII fragment of pUT322 (CAYLA) into the 4258-bp blunt *Hin*dIII fragment of pKAN-DIR (Friedman *et al.* 1995).

### Mutagenesis and consensus sequence alignments

The revertible selectable marker method was used for mutagenesis (Deng and Nickoloff 1992). Oligonucleotides were purchased from Gibco BRL. The sequences of the mutations m1-10 are given in Figure 3A. The sequences of the mutations m11, m12, and ∆NsiI are given in Figure 3D. The sequences of the DNA unwinding elements (DUE) mutations corresponding to the m5-m7 positions were m5DUE (ATCAAGTACT; data not shown), m6DUE (ATAGACGTCA; Supporting Information, Figure S2), and m7DUE (ATTAATAGT; Figure S2). Correct transformants were identified by restriction digestion screening for incorporation of a restriction site (*Xho*I for m1-10; *Sca*I for m5DUE; *Aat*II for M6DUE; *Ase*I for m7DUE), and the mutations were confirmed by sequencing. The success rate of the mutagenesis was generally approximately 60%.

Consensus matches for the binding sites of Abf1p and Rap1p were identified by the use of the Intelligenetics program Seq. The parameters used were as follows: GapPenalty = 1, GapSizePenalty = 0.3333, MinAlign = 4, MisMatchPenalty = 0, OverLap = 33.3%, SegmentSize = 30, Expect = 10, LoopOut = 3, and MinMatch = 5. Sequences were entered using the Intelligenetics program GENED. Computer analysis of DUE was performed using VooDUE (M. K. Raghuraman, unpublished results), a Macintosh version of the Thermodyne program (Natale *et al.* 1992, 1993). It calculates the energy of unwinding of the DNA duplex in the same manner as the Thermodyne program.

### Two-dimensional (2-D) gel electrophoresis

Samples for 2-D gels were collected at 1.5-min intervals for 33 min after release of the cells into S phase, then pooled. Cell synchrony and release were performed as described (Brewer *et al.* 1992). DNA was prepared as described (Huberman *et al.* 1987; Brewer *et al.* 1992). Determining the direction of replication fork movement by 2-D gels involved an in-gel digestion as described (Friedman and Brewer 1995). For every construct, the west half of the plasmid was analyzed. The blots were hybridized to a radioactively labeled 1185-bp *Bgl*I-*Nde*I fragment of pBR322 and analyzed on an InstantImager (Packard) for quantitation.

### Determination of replication time

Density shift experiments on the strains analyzed in Figure 4B and Figure 5E were respectively performed in strains RM14-3a and KK14-3a (a URA+ version of RM14-3a) as previously described (McCarroll and Fangman 1988). The data were analyzed using an InstantImager or phosphorimager (Packard or BioRad, respectively). The comparative hybridization experiments were performed essentially as described (Friedman *et al.* 1995). Strain 3xRZeoDIR containing pN&Sdir or pN&SDdir (described in the section *Results*) was incubated for 1.25 generations in α-factor. Upon arrest in the G1 phase, an uninduced sample was taken and galactose was added to 2%. Excision of the ARS-less cassette was allowed to proceed for four hours. Glucose was then added to 2% and the cells were released from α-factor arrest at 37°, the restrictive temperature for *cdc7^ts^*. Upon *cdc7* arrest, a sample was taken, and the cells were returned to 23° to allow entry into S phase. Samples were taken at 4-min intervals throughout S (60 min). The DNA was prepared by the “Smash and Grab” procedure (Hoffman and Winston 1987) and digested with *Bam*HI and *Eag*I. The samples were run on a 0.7% agarose gel, blotted onto Hybond (Amersham) nylon membrane, and hybridized to a *Bam*HI-*Eag*I fragment of pBR322 labeled with ^32^P. Quantification of the blots was performed on a PhosphorImager (Molecular Dynamics) using ImageQuant software.

## Results and Discussion

### Identifying the target of the bias determinant

We have previously shown that in the plasmid pN&S (Figure 1A), *ARS1^S^* is used four times more frequently than is *ARS1^N^* and that the preferred use of *ARS1^S^* is dependent on sequences residing in the 3′ end of the *URA3* gene (Brewer and Fangman 1994). Furthermore, this biased use of *ARS1^S^* is not dependent on *URA3* transcription.

Initiation bias in this two-*ARS1* plasmid was determined by examining the direction in which replication forks move through a restriction fragment that lies between the two copies of *ARS1* (Figure 1A). If *ARS1^S^* is active, forks move northward; if *ARS1^N^* is active, forks move southward. Replication intermediates of the *Bgl*II fragment from the west half of the plasmid generate an arc of Y molecules on standard 2-D gels with replication forks from *ARS1^N^* producing the same arc of Ys as forks from *ARS1^S^* (Brewer and Fangman 1994). To distinguish the two sets of replication intermediates, we included an in-gel restriction digestion with *Pst*I before the second dimension of electrophoresis and probed for the larger *Pst*I-*Bgl*II fragment. Ys created by forks emanating from *ARS1^S^* create an arc that rises from the 1N spot, whereas Ys from *ARS1^N^* create an arc that rises from a position displaced from the 1N spot (Figure 1B; Friedman and Brewer 1995). The amount of signal in each arc of Ys is quantified (Figure 1B) to yield the percentage of molecules replicated by forks from *ARS1^S^*—values greater than 50% indicating biased initiation from *ARS1^S^*. The original two-ARS plasmid, pN&S (Figure 1C), shows *ARS1^S^* initiation 75.2% of the time based on the values obtained from five different cultures (*ARS1^S^* initiation values of 71%, 74%, 74%, 77%, and 80%). These values provide a baseline for comparison with other constructs. Because the labor involved in each gel experiment limits the sample sizes that can reasonably be obtained, in testing other constructs described hereafter, we have chosen to be conservative in deciding whether biased initiation is significantly different from that of pN&S. Thus, we conservatively conclude that *ARS1^S^* initiation percentages falling outside the range of 68.1–82.3% (75.2 ± 7.1%; Students *t*-test 99% confidence interval) are significantly different from pN&S at the 1% level.

We first determined the target of the bias determinant residing at the 3′ end of the *URA3* gene. This initiation bias could be due to an effect on the activation of *ARS1^N^* or on *ARS1^S^*. To determine which copy of *ARS1* was being affected by the 3′ *URA3* sequences, we made two derivatives of pN&S, each having one of the two copies of *ARS1* inverted compared to its original orientation. ARS sequences are inherently asymmetrical because they are composed of discrete A and B elements: the three B elements in *ARS1* lie on the 3′-side of the T-rich strand of the 17-bp AT-rich ARS consensus sequence (A element) (Figure 1A; Newlon 1996). Because of this asymmetry, we reasoned that the orientation of one of the copies of *ARS1* with respect to the bias determinant might be important for the initiation bias.

We found that inversion of *ARS1^N^* in pN&S fails to reduce the bias (74% *vs.* 83%; compare Figure 1, C and D), whereas inversion of *ARS1^S^* reduces it substantially (74% *vs.* 56%; compare Figure 1, C and E). Because the endpoints of the *ARS1^S^* inversion do not include the sequences required for the initiation bias (described below), the reduction of the initiation bias can be attributed to the failure of the bias determinant to act on the ARS when the ARS is in inverted orientation and not to the disruption of the sequence causing the bias. These data indicate that the primary target of the bias determinant is *ARS1^S^* rather than *ARS1^N^*.

### Location and structure of the bias determinant

**Figure 2:**
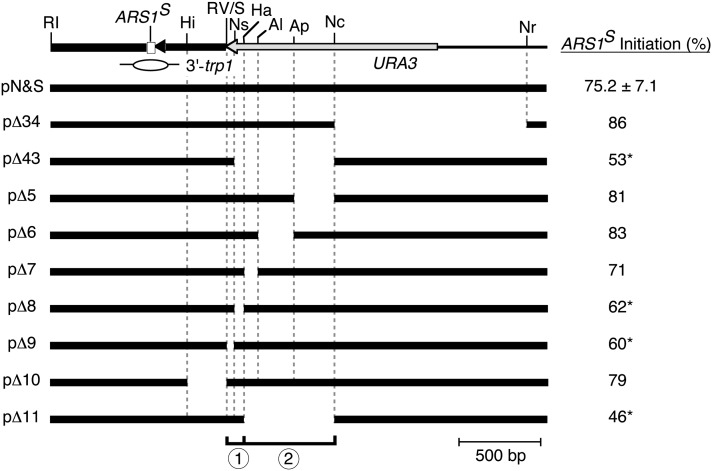
Deletion analysis of 3′ *URA3* sequences important for the initiation bias. Top, A map of the southern region of pN&S including *ARS1^S^* and the *URA3* gene is shown. A bubble indicates the position of *ARS1^S^*. Values for percent initiation at *ARS1^S^* are given for pN&S and nine deletion derivatives. Values that are significantly reduced compared with the value obtained for pN&S on five separate experiments are indicated by (*). Deletion constructs are as follows: (pΔ34) ∆*Nco*I-*Nru*I (Brewer and Fangman 1994); (pΔ43) ∆*Nsi*I-*Nco*I (Brewer and Fangman 1994); (pΔ5) ∆*Apa*I-*Nco*I; (pΔ6) ∆*Alw*NI-*Apa*I; (pΔ7) ∆*Hae*III-*Alw*NI; (pΔ8) ∆*Nsi*I-*Hae*III; (pΔ9) ∆*Sma*I-*Nsi*I; (pΔ10) ∆*Hin*dIII-*Eco*RV; (pΔ11) ∆*Hae*III-*Nco*I. Restriction sites are as follows: (Al) *Alw*NI; (Ap) *Apa*I; (RI) *Eco*RI; (RV) *Eco*RV; (Ha) *Hae*III; (Hi) *Hin*dIII; (Nc) *Nco*I; (Nr) *Nru*I; (Ns) *Nsi*I; (S) *Sma*I.

Determination of the initiation bias in the two-ARS plasmid provides a relatively simple assay for a mutational analysis of this element. Previous observations showed that deletion of the *Nco*I-*Nru*I fragment, which included the 5′ half of *URA3* (plasmid pΔ34), does not eliminate the preferential use of *ARS1^S^*, but that deletion of the *Nsi*I-*Nco*I fragment from the 3′ end of *URA3* (plasmid pΔ43) results in equivalent use of both copies of *ARS1* (Figure 2; Brewer and Fangman 1994). To further localize the early determinant, four deletions (plasmids pΔ5, pΔ6, pΔ7, and pΔ8) that remove sections of the *Nsi*I-*Nco*I sequence were constructed using convenient restriction sites, and tested for origin bias. Deletion of the 169-bp *Apa*I-*Nco*I fragment (pΔ5), the 302-bp *Alw*NI-*Apa*I fragment (pΔ6), or the 113-bp *Hae*III-*Alw*NI fragment (pΔ7) had no significant reduction of the bias. However, the 34-bp *Nsi*I-*Hae*III fragment contributes substantially to the bias because its deletion (pΔ8) significantly reduced the bias (Figure 2).

To examine a possible role of sequences immediately to the left of the *Nsi*I-*Hae*III fragment, we made a 56-bp *Sma*I-*Nsi*I deletion (plasmid pΔ9). This deletion greatly reduced the bias (Figure 2), showing that these deleted sequences also play a major role. Further deletion to the left via the removal of the 228-bp *Hind*III-*Sma*I fragment, including a portion of the *TRP1* sequences (plasmid pΔ10), had little effect on the initiation bias (Figure 2). On the basis of these results, plasmid sequences that contribute substantially to initiation bias appear to lie largely within the 90-bp *Sma*I to *Hae*III sequence in the *URA3* gene (Figure 2, bracket 1). However, despite finding little effect of deletions ∆5, ∆6, and ∆7, which together span the 584-bp *Hae*III-*Nco*I fragment to the right of the *Sma*I-*Hae*III section, deletion of the entire *Hae*III-*Nco*I fragment (plasmid pΔ11) eliminated the origin bias (Figure 2). Because no single internal deletion in this region (pΔ5, pΔ6, and pΔ7) dramatically reduced the initiation bias, there appear to be functionally redundant contributions to origin bias by the *Hae*III-*Nco*I region. In summary, sequences within the *Hae*III-*Nco*I fragment (Figure 2, bracket 2), along with those in the *Sma*I-*Hae*III fragment, contribute to biased origin use. Due to the apparent complexity of sequences within the *Hae*III-*Nco*I region, we focused our further studies on the unique 90 bp fragment (Figure 2, bracket 1).

**Figure 3:**
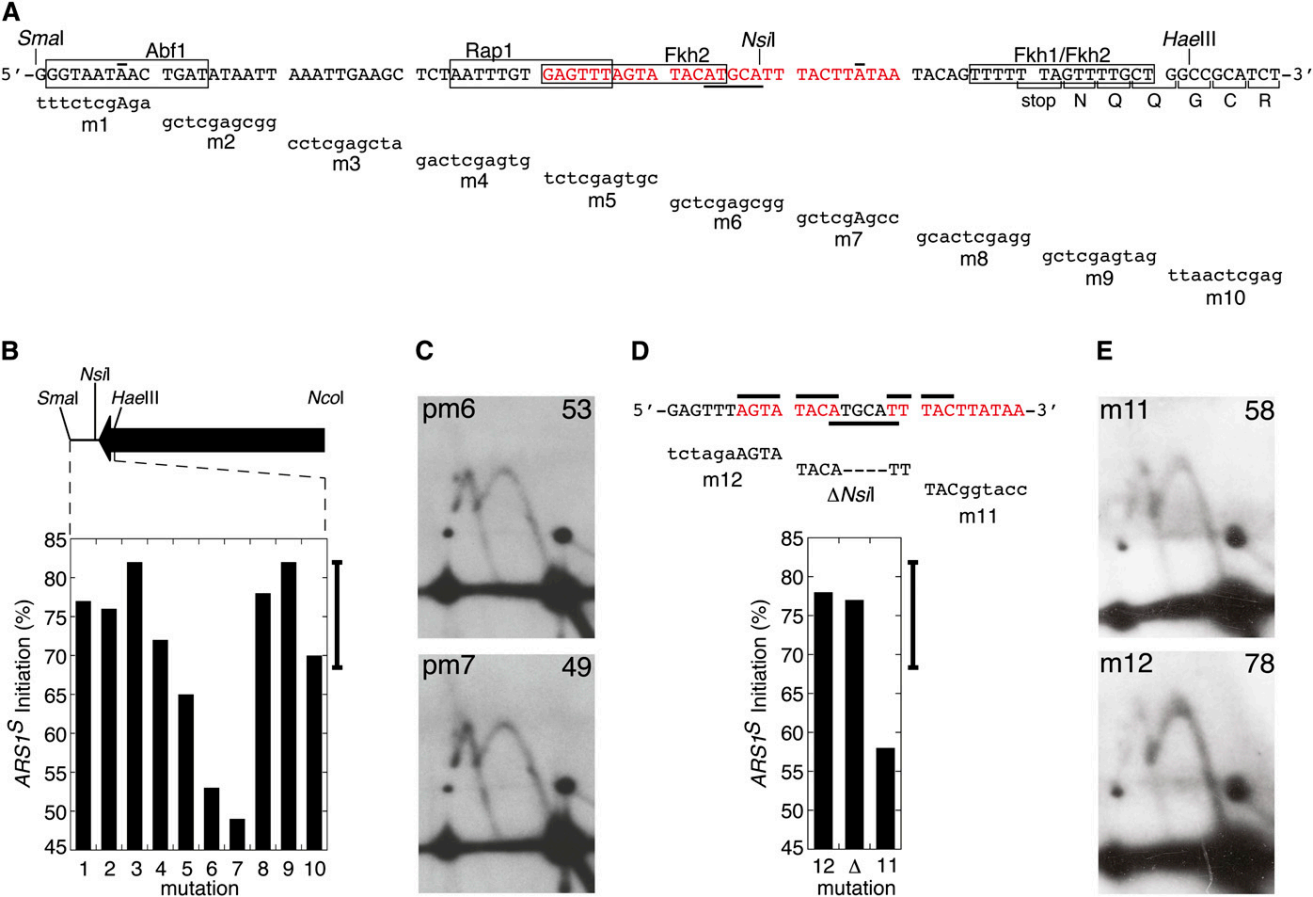
Scanning mutagenesis analysis for sequences involved in initiation bias. (A) The wild-type sequence of the *Sma*I-*Hae*III fragment of *URA3* is shown in capital letters. Sequences of the mutations are given in lowercase letters beneath the wild-type sequences they replace. The two overlined nucleotides were not changed by the mutagenesis. The boxes identify 11 of 13 bp matches to Rap1 or Abf1 consensus binding sites as well as matches to Fkh1 and Fkh2. The 3′ end of the *URA3* coding sequence is indicated by the single letter amino acid code. The locations of the *Sma*I, *Nsi*I, and *Hae*III restriction enzyme sites are indicated. Sequences that when mutated significantly reduce the *ARS1^S^* bias are indicated in red. (B) Values for percent initiation at *ARS1^S^* are plotted for the 10 mutations that span the *Sma*I-*Hae*III fragment. The bar on the right of the graph indicates a 99% confidence interval extending on both sides of the mean percent initiation at *ARS1^S^* for pN&S based on five separate experiments. Values that fall outside of this range are considered significantly different from pN&S at the 1% level. The approximate locations of the mutations relative to the 3′ half of the *URA3* gene (arrow) are shown. (C) Fork-direction gel analysis of mutants m6 and m7 as examples of the mutations that eliminate the origin bias. (D) The wild-type sequence corresponding to the sequences of the *URA3* fragment that were mutated in m5, m6, and m7 are shown in capital letters. Sequences of three different mutations (m12, ∆*Nsi*I, m11) are given in lowercase letters beneath the wild-type sequences they replace. Deleted bases are depicted by a dash. The overlined nucleotides were not changed by the mutagenesis while the underlined nucleotides mark the *Nsi*I site. Sequences important for the bias are indicated in red letters. Percent initiation at *ARS1^S^* is indicated as bar graphs for the three mutations, m11, m12, and ∆*Nsi*I. (E) Fork direction 2-D gels of mutants m11 and m12 (see panel D for sequences). Percent initiation at *ARS1^S^* is indicated in the upper right corners.

To further determine which sequences within the 90-bp *Sma*I-*Hae*III fragment (Figure 2, bracket 1) contribute to bias determinant activity, this fragment was examined by scanning site-directed mutagenesis. Typically, 10-bp changes were made (one mutation, m7, was a 9-bp change) (Figure 3A). Because an *Xho*I restriction site was also included for screening purposes two base pairs of the 90 bp examined were not changed by the mutagenesis (Figure 3A, overlines). Analysis of *ARS1^S^* initiation percentage for the 10 mutants (Figure 3B) revealed that two of them, m6 and m7 (Figure 3C), exhibited dramatic reductions in the initiation bias whereas a third mutant, m5, showed a slightly reduced initiation bias. The other seven mutations gave initiation biases that were not significantly different from that of the original two-ARS plasmid (Figure 3B, vertical bracket). Therefore, through scanning mutagenesis we identified a 29-bp sequence (Figure 3A, red) within the *Sma*I-*Hae*III fragment that contains bases important for origin bias.

To further characterize bases that are essential for the origin bias, three plasmids were constructed to have a 6-bp mutation on either the 5′ or the 3′ ends of the 29-bp sequence or containing a central 4-bp deletion. These plasmids were also analyzed via fork direction 2D gel analysis (Figure 3, D and E). Although the six bases on the 3′ end of the fragment are essential for origin bias, the six bases on the 5′ end and the four bases that were centrally deleted are not. These data result in the identification of a 19-bp bipartite sequence that is essential for origin bias (Figure 3D, red). The essential 19 bases are consistent with the results obtained for plasmids pΔ8 and pΔ9 in our deletion analysis (see Figure 2). Interestingly, our data suggest that although the identified 19 bases are essential for the bias they are not sufficient to cause the bias, requiring the presence of sequences within the broader 584-bp *Hae*III-*Nco*I fragment.

### The bias determinant advances *ARS1^S^* origin firing time relative to *ARS1^N^*

The observation that the bias determinant at the 3′ end of *URA3* is influencing *ARS1^S^* (Figure 1) without preferentially affecting its efficiency relative to that of *ARS1^N^* (Brewer and Fangman 1994) led us to the hypothesis that the determinant causes the bias by advancing *ARS1^S^* activation time. Because *ARS1^N^* is used approximately 20% of the time it suggests that, even if the *ARS1^S^* is usually activated earlier than is *ARS1^N^*, there is some overlap in the distribution of their activation times. It seems likely, therefore, that the difference between their mean times of activation could differ by a small increment, perhaps by as little as one minute. We tested this origin timing hypothesis using three different experimental approaches.

#### Comparison of 3′URA3 sequence and a centromere:

If the bias determinant were to impart early activation to *ARS1^S^* we would expect that other DNA elements, such as centromeres, that are known to advance origin timing would produce a similar bias when placed in the context of the two-ARS plasmid. We would also predict that a centromere placed near the opposite copy of *ARS1* would diminish or neutralize any bias that would be imposed by the 3′ *URA3* bias element.

**Figure 4:**
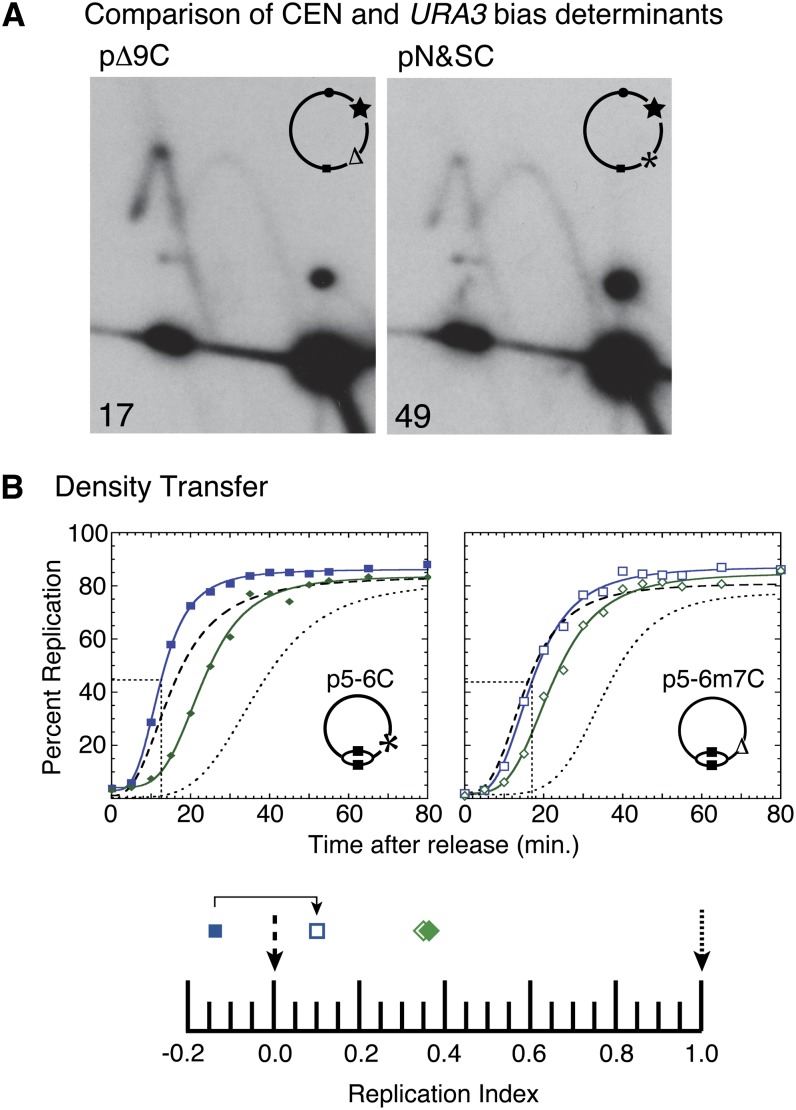
Replication kinetics of *ARS1* in the presence and absence of *URA3* sequences that cause biased origin activation. (A) Fork direction 2-D gel analysis was performed on plasmids in which the bias determinant is present (pN&SC, illustration: *) or absent (pΔ9C, illustration: ∆). Both plasmids contained a centromere near *ARS1^N^* (illustration: star). Percent initiation at *ARS1^S^* is indicated in the bottom left corners. (B) Replication kinetics of stable plasmids containing only *ARS1^S^* from dense isotope transfer experiments. The *T*_rep_ value for each plasmid is shown as a dotted line intersecting the X-axis. Upper left: replication kinetics for a plasmid (p5-6C) containing the wild-type 3′ *URA3* sequence that results in preferential use of *ARS1^S^* in the original pN&S plasmid (solid blue squares). Upper right: replication kinetics for a plasmid (p5-6m7C) with a 9-bp substitution mutation in the 3′ *URA3* sequence that results in equivalent origin use in the original pN&S plasmid (open blue squares). The kinetics of plasmid replication are compared with a sequence that replicates early in S phase, *ARS305* (dashed); in mid-S phase, chromosomal *ARS1* (filled or open green diamonds); and late in S phase, R11 (dotted). The presence (*) or absence (∆) of the wild-type 3′ *URA3* sequence is shown on the illustrations of the plasmids. Replication indices for the plasmids and *ARS1* in each strain are shown in the lower panel.

We first asked whether a centromeric sequence could produce a similar bias as the 3′ *URA3* sequence in the context of pN&S. To do so, we used a derivative of pN&S that lacks the sequence necessary for 3′ *URA3* dependent bias and therefore shows no ARS bias (pΔ9, see Figure 2). Integration of a centromere close to *ARS1^N^* in this plasmid (pΔ9C) resulted in a marked bias toward *ARS1^N^* use, indicating that sequences that are known to advance origin activation time in chromosomal DNA can in fact impose an origin use bias in pN&S (Figure 4A, left). Although a centromere’s ability to advance origin activation time is bidirectional, the centromere also has a greater timing effect on closer origins than on more distant ones (Pohl *et al.* 2012). Therefore, it is conceivable that in the context of the two-ARS plasmid the centromere is advancing the activation time of both *ARS1* copies, with *ARS1^N^* being advanced to a greater extent. Insertion of the centromere near *ARS1^N^* can also neutralize the bias imparted by the 3′ *URA3* sequence: *ARS1^S^* and *ARS1^N^* were equally used on a plasmid harboring both a centromere nearest to *ARS1^N^* and the intact 3′*URA3* sequences (Figure 4A, right). The equal usage of the two *ARS1* copies can be interpreted as *ARS1^S^* undergoing activation at a time similar to *ARS1^N^*, which is replicated early in this situation due to the nearby centromere. These data suggest that the 3′ *URA3* sequences are capable of advancing origin activation time.

#### The bias determinant promotes early ARS1^S^ activation:

To further test whether the bias determinant promotes the earlier activation of *ARS1^S^*, replication times were determined for plasmids that contain only *ARS1^S^*, either with or without a functional bias determinant. If the bias determinant were causing a delay in the activation of *ARS1^N^*, then deletion of the bias determinant should have no effect on the time of activation of *ARS1^S^* and the two plasmids should replicate at the same time. If, instead, the bias determinant were advancing the activation time of *ARS1^S^* then the plasmid with the bias determinant should replicate earlier than the one without it. Plasmid p5-6C is identical to the two-*ARS1* plasmid, pN&S, with two exceptions: (1) the 1.45-kb *EcoRI* fragment containing *ARS1^N^* has been removed, and (2) a 1.1-kb *CEN5* fragment has been cloned into the *BamHI* site. A second plasmid, p5-6m7C, is identical to p5-6C except that it contains a 9-bp substitution mutation, m7, which eliminates the initiation bias in the two-ARS plasmid (Figure 3C). Because *CEN5* is present at the same location in both p5-6C and p5-6m7C, any timing effect that *CEN5* would impart on *ARS1* would be equal in both plasmids. Integration of *CEN5* does not interfere with detecting the influence of the 3′ *URA3* sequence on *ARS1^S^* (Figure 4A) and was necessary to achieve the efficient plasmid maintenance required for density transfer experiments.

Plasmids p5-6C and p5-6m7C were transformed separately into *S. cerevisiae* and their times of replication were determined in parallel cultures using a modified version of the Meselson-Stahl density shift experiment with synchronized cultures (McCarroll and Fangman 1988). Cultures of cells with these two different plasmids have the same population doubling times. The percent replication of p5-6C and p5-6m7C was calculated for each sample and plotted with respect to time (Figure 4B). The time of replication (*T*_rep_) for each locus was calculated as the time at which that locus reached half maximal replication. *ARS305*, one of the earliest known origins, and R11, a late replicating fragment on chromosome V, were used as timing standards. To facilitate comparison between cultures, these *T*_rep_ values were converted to replication indices (Friedman *et al.* 1996) by assigning *ARS305* a replication index (RI) of 0 and R11 a RI of 1.0 (Figure 4B, lower panel). Most other genomic loci have a RI between 0 and 1.0. The *T*_rep_ values for the two plasmids and a sequence immediately flanking chromosomal *ARS1* were then converted to RIs corresponding to the fraction of the *ARS305*-R11 interval elapsed when the *T*_rep_ for each locus was obtained. p5-6C (RI = −0.14) replicated 3−4 min earlier than did a chromosomal fragment containing the early replicating origin, *ARS305*, whereas p5-6m7C (RI = 0.1) replicated at the same time as *ARS305* (Figure 4B). As expected, the replication indices for chromosomal *ARS1* fragments were similar between the two strains (RI = 0.35 and 0.36). These results indicate that the bias determinant acts positively on the *ARS1^S^* origin to advance its time of activation.

Consistent with these results, the 3-min timing advantage that the bias determinant imparts on *ARS1^S^* was recapitulated using a site-directed recombinase to split plasmid pN&S into a south half and a north half *in vivo*, allowing the firing time of *ARS1^N^* and *ARS1^S^* to be directly compared within the same cell (Figure S1). Together, the results of these three assays indicate that *URA3* sequences are influencing replication initiation time on the two-ARS plasmid by advancing *ARS1^S^* activation time, and that this difference in initiation time can explain the initiation bias. The 3- to 4-min advancement in activation time caused by the 3′*URA3* sequence explains how two equally efficient ARSs can exhibit large differences in levels of activation when placed in competition on a single plasmid molecule.

### The bias determinant can regulate origin activation time over a distance of at least 1 kb

**Figure 5:**
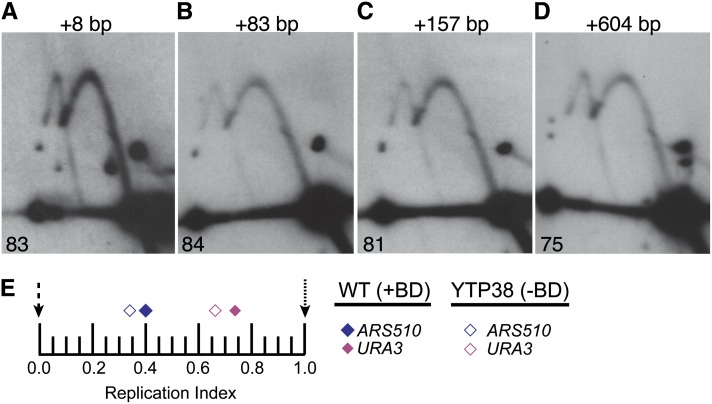
Determining distances over which the bias determinant can function. (A-D) Fork direction 2-D gel analysis on pN&S plasmids that have various lengths of sequence inserted within the *Nco*I-*Sma*I fragment, between the bias determinant and *ARS1^S^*. The amount of sequence that was inserted for each construct is indicated above each image. Percent initiation at *ARS1^S^* is indicated in the lower left corners. (E) Replication indices for chromosomal *URA3* (pink) and *ARS510* (blue) in wild-type (WT) cells harboring the bias determinant at its native location (closed diamonds) and in cells lacking the bias determinant (open diamonds) are plotted. *ARS607* (black dashed arrow) and R11 (black dotted arrow) were used as early and late timing standards, respectively. In the WT strain, *URA3* and *ARS510* had replication indices of 0.73 and 0.4, respectively. In the strain lacking the bias determinant (YTP38), *URA3* had a replication index of 0.66, whereas the replication index for *ARS510* was 0.34.

Other *cis*-acting timing determinants that have been identified can affect replication timing over great distances. Centromeres advance origin activation time over a distance of 20 kb (Pohl *et al.* 2012; Natsume *et al.* 2013), whereas telomeres delay the activation time of origins over about 30 kb (Ferguson *et al.* 1991). The activation times of two origins on chromosome XIV, *ARS1412*, and *ARS1413*, are delayed by cryptic sequences that reside up to 6 kb away (Friedman *et al.* 1996). The forkhead transcription factors, Fkh1 and Fkh2, are capable of advancing origin activation time over a distance of at least 500 bp (Knott *et al.* 2012). We therefore sought to determine the distance over which the *URA3* timing determinant can act. An 8-bp *Xho*I linker was inserted at the *Sma*I/*Eco*RV junction between *ARS1^S^* and the bias determinant to provide a cloning site for several subsequent insertions. This small insertion had no effect on the bias (Figure 5A). Insertion of either one or two copies of a 75-bp *Xho*I PCR lambda fragment at the XhoI site (Figure 5, B and C) or of a 604-bp lambda *Stu*I fragment at the *Sma*I/*Eco*RV junction (Figure 5D) also had no effect on the bias. Because the distance from the *Sma*I/*Eco*RV junction to the ACS of *ARS1^S^* is 465 bp, these data indicate that the bias determinant can function over a distance of at least 1069 bp.

An alternative, though unlikely, interpretation of this experiment is that the bias determinant is not able to function over this distance and that instead, the lambda sequences are promoting an initiation bias of their own. To eliminate this possibility, the bias determinant was compromised in the pN&S+604 bp construct by an *Nco*I-*Nsi*I deletion (see pΔ43 in Figure 2). This construct exhibits no bias (data not shown), indicating that the lambda sequences are not capable of conferring a bias on their own.

At its native location *URA3* resides at a distance from its neighboring origins that is ideal to test the outer limits over which the bias element can function. In the genome, *URA3* is flanked by two efficient early activating origins, *ARS508* and *ARS510*, that are located at respective distances of 21.9 and 28.8 kb from *URA3* (Nieduszynski *et al.* 2007). However, only *ARS510* is in the correct orientation to have its activation time regulated by the bias determinant. Therefore, we expect that if the bias determinant regulates origin activation at its native location its effect would be on *ARS510*. To determine whether the bias determinant influences replication at its native genomic location, we performed a density shift experiment on cells in which the chromosomal copy of the 3′ *URA3* bias element had been removed (Figure 5E). Cells lacking the bias determinant replicated both the *URA3* gene and *ARS510* similar to control cells, suggesting that the bias determinant does not detectably regulate the activation time of flanking origins at its native location. These results could also be obtained if the bias determinant was effective only on plasmids and incapable of advancing origin activation time in the context of a linear chromosome. However, we can rule out this possibility because the bias effect persists when pN&S is integrated into the chromosomal *ARS1* locus (Brewer and Fangman 1994).

Taken together, these experiments indicate that the *URA3* timing determinant can act over a distance of at least 1 kb but not over a distance of tens of kilobases. As more sequences that are capable of regulating origin activation time are identified, it will be interesting to determine more precise limits or boundaries for each given regulator.

### DNA binding motif search of the bias determinant

The effects of deletions in the 3′ *URA3* region and of the inversion of *ARS1* on bias activity indicate that the *ARS1* element and the bias determinant are separate entities. Does the bias determinant communicate with the ARS through the binding of specific proteins to the bias determinant? On the basis of our observation that the 3′ end of *URA3* consists of a complex, multipartite timing determinant, we were aware that identifying any *trans*-acting factor(s) mediating the effect of this determinant might be challenging, for example, that redundancy introduced by multiple *trans*-acting factors interacting with the determinant might complicate the analysis. Nevertheless, finding that at least one component of the determinant could be narrowed to a small (19−23 bp) element encouraged us to embark on a search for proteins such as transcription factors that might mediate the timing effect by binding to that sequence. To this end, we first considered proteins known to influence origin activity. *ARS1* contains a binding site for the transcription factor Abf1 in element B3 that contributes to ARS activity (Marahrens and Stillman 1992), and it has been demonstrated for *ARS121* that a second Abf1 binding site, outside of the B region, contributes to maintenance of an *ARS121* plasmid under some culture conditions (Walker *et al.* 1989). In addition, a binding site for a different transcription factor (Rap1) was shown to substitute for the Abf1 site at *ARS1* (Marahrens and Stillman 1992). Although the mechanism by which transcription factors enhance ARS function is not known, these observations suggest that transcription factor binding might contribute to the bias observed in pN&S. There is a potential binding site for Abf1 and one for Rap1 in the 90 bp *Sma*I-*Hae*III fragment of the bias element region. However, neither of these potential sites overlaps the 23 bp essential core element identified by scanning mutagenesis (Figure 3), indicating that these transcription factors are not primarily responsible for the origin initiation bias. Given the complexity of the bias determinant, however, a possible redundant role for Abf1 and Rap1 cannot be excluded.

The forkhead transcription factors, Fkh1 and Fkh2, recently have been shown to influence the initiation time of some origins (Knott *et al.* 2012), raising the possibility that these transcription factors might have some role in the observed bias determinant. Furthermore, there are potential binding sites for Fkh1 and Fkh2 close to or partially overlapping the core bias element (Figure 3A). However, these binding sites do not overlap with all sequences that were shown to be crucial for the bias (see m11, Figure 3D), suggesting that Fkh1 and Fkh2 are likely also not involved in the observed origin initiation bias. Consistent with this logic, preliminary chromatin immunoprecipitation studies performed by the Fox lab indicate that Fkh1 does not bind this sequence at its native chromosomal location (T. Hoggard and C. Fox, personal communication).

**Figure 6:**
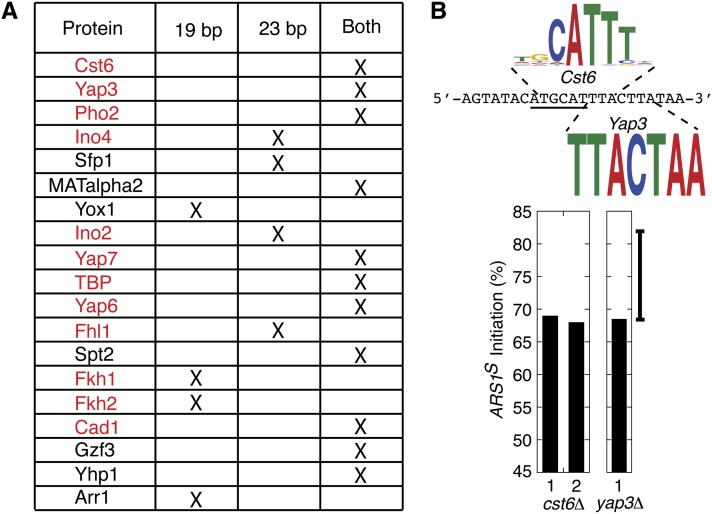
Motif search of sequences important for the bias determinant. (A) Both the 23-bp (+TGCA) and the bipartite 19-bp (−TGCA) sequences were run through the motif identification tool TOMTOM (Gupta *et al.* 2007). Candidate proteins that were identified for each sequence are listed in order of significance (top to bottom) with proteins common to both searches in the last column. Transcription factors are indicated by red text. (B) The WT sequence corresponding to the 23 bps (+TGCA) that are required for the bias is indicated by small black capital letters. The *Nsi*I site is underlined. The logos for Cst6 and Yap3 binding motifs are indicated above or below the corresponding sequence. Percent initiation at *ARS1^S^* on pN&S in cells lacking either *CST6* or *YAP3* are indicated as bar graphs.

Because the obvious candidates are likely not responsible for the bias, we embarked on a more systematic search for *trans*-acting factors that might bind to the bias determinant. Both the essential 23-bp sequence and the 19-bp sequence (with the four central bases deleted) were run through the motif comparison tool TOMTOM (Gupta *et al.* 2007). In both instances, binding motifs for multiple proteins were identified, many of which are specific to transcription factors (Figure 6A).

The top hit in both searches was the binding motif for the transcription factor Cst6 (Figure 6). Of particular interest was the finding that while the Cts6 binding motif overlaps the *Nsi*I site, the motif would be restored in the four-base (TGCA) deletion (Figure 6B). The next candidate on the list was the transcription factor, Yap3. Like all other proteins on the list, the Yap3 consensus binding motif only overlapped with part of the sequence that was found to be essential for the bias and like many of the other proteins Yap3 has an imperfect target-site match (Figure 6B). To determine whether either of the top two hits (Cst6 or Yap3) was responsible for the bias, plasmid pN&S was transformed into cells lacking either *CST*6 or *YAP3* and analyzed for the presence or absence of the bias (Figure 6B). As in WT cells, an initiation bias for *ARS1^S^* was observed for pN&S indicating that neither Cst6 nor Yap3 binding is responsible for the bias.

Because we have excluded the “top” two candidates from our motif search, a systematic approach will need to be taken to analyze the remaining proteins that are potentially involved. However, from these studies, we have concluded that given the large number of potential binding candidates, the degeneracy of transcription factor binding sites, the possibility of redundancy at the bipartite site, and the labor involved in testing each candidate, an approach other than gene knockouts will be required to identify the putative *trans*-acting factors that regulate this initiation timing determinant. Furthermore, it is possible that none of the candidate proteins identified in our motif search are uniquely involved in creation of the bias. This possibility is supported by the observation that none of the motifs for the remaining candidates span the entire 23-bp motif that is essential to the bias. It is worth noting that the percentages of *ARS1^S^* usage in both *CST6* and *YAP3* knockout strains are either very close to or slightly below the bottom of the confidence interval. Though these data were not deemed significantly different from pN&S, it would be interesting to determine how a double knockout of *CST6* and *YAP3* would affect *ARS1^S^* bias. The observation that double knockouts of transcription factors can present adverse phenotypes such as pseudohyphal growth (Knott *et al.* 2012) adds to the complexity of such studies.

Finally, we considered the possibility that bias determinant acts directly via some intrinsic property of its own, not necessarily mediated by the binding of *trans*-acting factors. The B2 element of *ARS1* is part of a DNA unwinding element (DUE), which is believed to enhance ARS function through its inherent helical instability (Natale *et al.* 1992, 1993). Computer analysis of the *Sma*I-*Hae*III fragment of pN&S shows that it is part of a DUE (Natale *et al.* 1992, 1993). Does this DUE contribute, autonomously or in conjunction with the binding of a protein, to earlier activation of *ARS1^S^*? We made several site-directed mutations of the 19-bp core sequence, scrambling the sequence without altering the predicted helical stability (see the section *Materials and Methods*). These mutations did substantially reduce the origin bias (Figure S2). Therefore, the 19-bp sequence appears to contribute to origin bias in a way that depends on its specific sequence and not simply on its propensity to unwind. Although a role for this particular DUE at the 3′ end of the *URA3* gene in origin bias cannot be excluded, a DUE alone is not sufficient to advance the time of origin firing.

Other than our studies examining the bias determinant at its native location, the ability of this early timing determinant to influence other ARS elements has not yet been tested. The possible response of other ARS elements can be approached first, and most simply, by using the origin bias assay. However, it may be that response to the early determinant requires specific sequences within *ARS1*. If so, the sequence elements in *ARS1* that respond to the determinant could be identified by the use of mutagenesis of *ARS1^S^* in the context of the two-ARS plasmid assay. For any mutation that reduces the origin bias, it would be important to alter both ARSs in the plasmid identically to distinguish between mutations that reduce origin function from those that are specific to the origin’s ability to respond to the bias determinant. In addition, the bias assay with the two-ARS plasmid has the potential for providing a sensitive tool for characterizing other *cis*-acting factors involved in replication initiation timing control. For example, sequences on chromosome XIV have been shown to delay the time of activation of origins that they flank, and they have been shown to act on *ARS1* transplanted from chromosome IV (Friedman *et al.* 1996). These late determinants can delay origin initiation by 10 min or more, and might cause an even greater bias in the two-ARS plasmid than that conferred by the 3′ *URA3* early determinant.

## Supplementary Material

Supporting Information
