# Supplementary material for: A DNA Sequence Element That Advances Replication Origin Activation Time in Saccharomyces cerevisiae
Source: G3 (Bethesda). 2013 Nov 1;3(11):1955–63. doi: 10.1534/g3.113.008250 (PMC3815058; doi:10.1534/g3.113.008250)
Supplement: Supporting Information [file supp_3_11_1955__index.html]

A DNA Sequence Element That Advances Replication Origin Activation Time in Saccharomyces cerevisiae — Supporting Information 

# A DNA Sequence Element That Advances Replication Origin Activation Time in *Saccharomyces cerevisiae*

## Supporting Information for Pohl *et al.*, 2013

**Files in this Data Supplement:**

- Supporting Information - Figures S1-S2 (PDF, 872 KB)
- Figure S1 - *ARS1S* activation time analyzed by plasmid pop-out assay (PDF, 410 KB)
- Figure S2 - Examining the importance of a DUE at the bias determinant (PDF, 526 KB)
