# Supplementary material for: A DNA Sequence Element That Advances Replication Origin Activation Time in Saccharomyces cerevisiae
Source: G3 (Bethesda). 2013 Nov 1;3(11):1955–63. doi: 10.1534/g3.113.008250 (PMC3815058; doi:10.1534/g3.113.008250)
Supplement: Supporting Information [file supp_g3.113.008250_008250SI.pdf]

G3·Genes | Genomes | Genetics

A DNA Sequence Element that Advances Replication Origin Activation Time in *Saccharomyces cerevisiae*

Thomas J. Pohl[†,‡,1], Katherine Kolor[‡,§,1], Walton L. Fangman[‡], Bonita J. Brewer[†,‡], and M. K. Raghuraman[‡,2]

[1]Co-first authors

[2] Corresponding author: M. K. Raghuraman, Department of Genome Sciences, University of Washington, Box 355065 Foege Bldg., Room S-041, 3720 15th Ave NE, Seattle, WA 98195-5065 E-mail: raghu@u.washington.edu

Author affiliations:

[†]Molecular and Cellular Biology Program and [‡]Department of Genome Sciences, University of Washington, Seattle, Washington 98195 and [§]Office of Public Health Genomics, Centers for Disease Control and Prevention, Atlanta, Georgia 30329

DOI: 10.1534/g3.113.008250

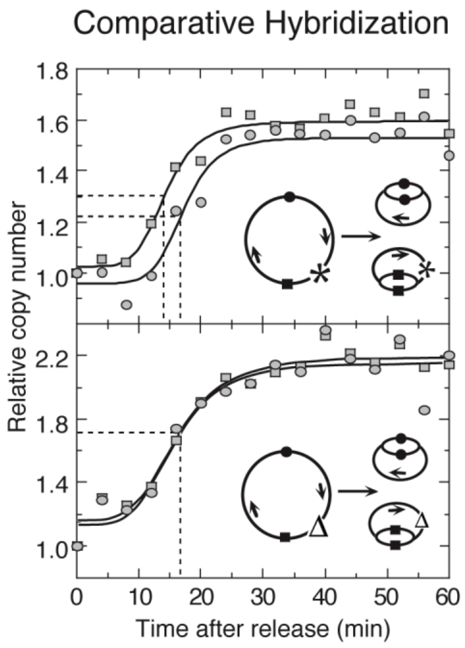

Figure S1 *ARS1*^{S} activation time analyzed by plasmid pop-out assay. The two plasmid copies of *ARS1* were separated from each other in vivo. Upper panel: separation from plasmid pN&Sdir. Lower panel: separation from plasmid pN&SΔ9dir derived from pN&Sdir by deleting the NsiI-NcoI fragment. Replication kinetics determined by the comparative hybridization method (Friedman et al. 1995) are plotted for each separated, single-ARS plasmid: plasmid pS' or pS'Δ (squares) and plasmid pN' (circles). Recombinase target sites are indicated by arrows on the cartoons and (Δ) indicates the NsiI-SmaI deletion of the bias element (*). The Trep value for each plasmid is shown as a dotted line intersecting the X-axis.

References cited

Friedman, K. L., M. K. Raghuraman, W. L. Fangman and B. J. Brewer, 1995 Analysis of the temporal program of replication initiation in yeast chromosomes. J Cell Sci Suppl 19: 51-58.

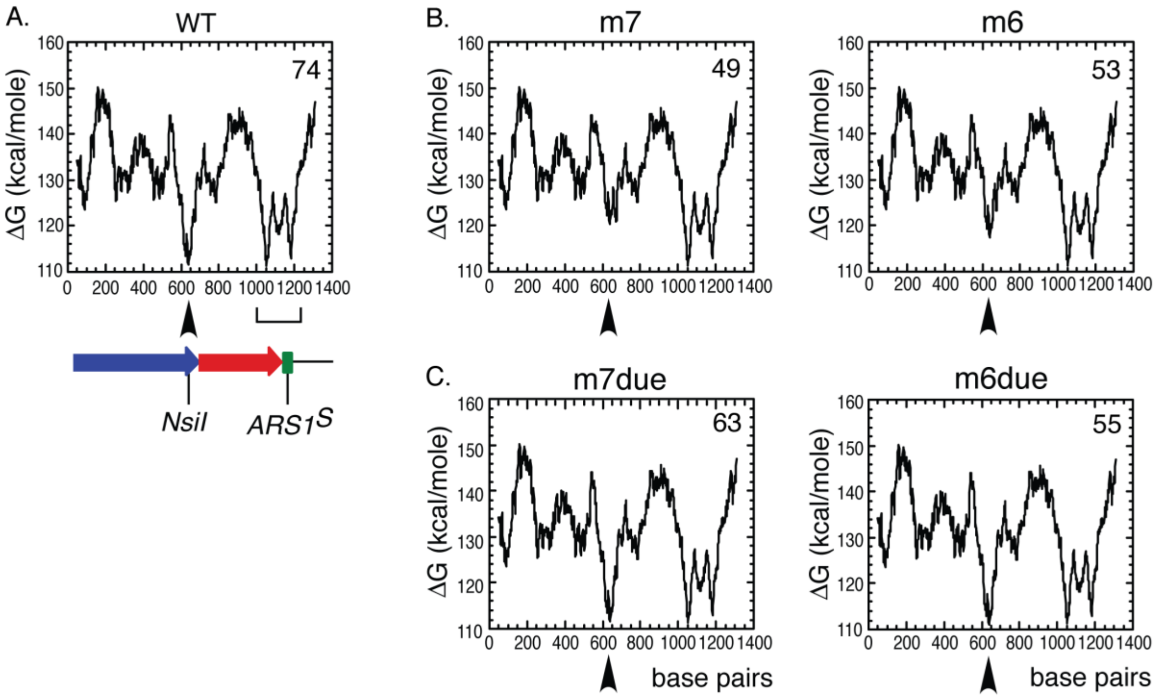

Figure S2 Examining the importance of a DUE at the bias determinant. Helical stability plots depicting the energy of unwinding (ΔG) of duplex DNA for the region containing the bias determinant (black arrow head). The percent of *ARS1^S* usage is depicted in the upper right corner. The cartoon depicts the sequence that was analyzed: the 3' end of *URA3* (blue arrow), the 3' end of *TRP1* (red arrow) and *ARS1^S* (green box). (A) Helical stability for the WT bias determinant and its surrounding region. The bracket indicates the *ARS1* DUE. (B) Helical stability for bias determinant mutants m6 and m7. (C) Helical stability for bias determinant mutants (see Materials and Methods) that maintain the presence of DUE but lack *ARS1^S* bias.
