# Supplementary material for: A DNA Sequence Element That Advances Replication Origin Activation Time in Saccharomyces cerevisiae
Source: G3 (Bethesda). 2013 Nov 1;3(11):1955–63. doi: 10.1534/g3.113.008250 (PMC3815058; doi:10.1534/g3.113.008250)
Supplement: Supporting Information [file supp_g3.113.008250_FigureS1.pdf]

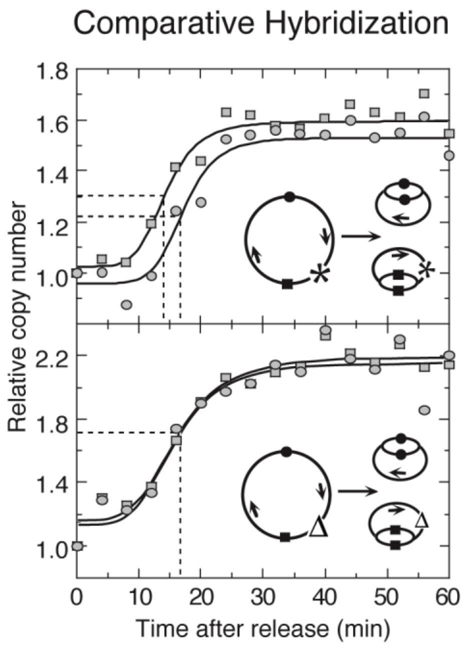

Figure S1 *ARS1*^{S} activation time analyzed by plasmid pop-out assay. The two plasmid copies of *ARS1* were separated from each other in vivo. Upper panel: separation from plasmid pN&Sdir. Lower panel: separation from plasmid pN&SΔ9dir derived from pN&Sdir by deleting the NsiI-NcoI fragment. Replication kinetics determined by the comparative hybridization method (Friedman et al. 1995) are plotted for each separated, single-ARS plasmid: plasmid pS' or pS'Δ (squares) and plasmid pN' (circles). Recombinase target sites are indicated by arrows on the cartoons and (Δ) indicates the NsiI-SmaI deletion of the bias element (*). The Trep value for each plasmid is shown as a dotted line intersecting the X-axis.

References cited

Friedman, K. L., M. K. Raghuraman, W. L. Fangman and B. J. Brewer, 1995 Analysis of the temporal program of replication initiation in yeast chromosomes. J Cell Sci Suppl 19: 51-58.
