# Supplementary material for: A DNA Sequence Element That Advances Replication Origin Activation Time in Saccharomyces cerevisiae
Source: G3 (Bethesda). 2013 Nov 1;3(11):1955–63. doi: 10.1534/g3.113.008250 (PMC3815058; doi:10.1534/g3.113.008250)
Supplement: Supporting Information [file supp_g3.113.008250_FigureS2.pdf]

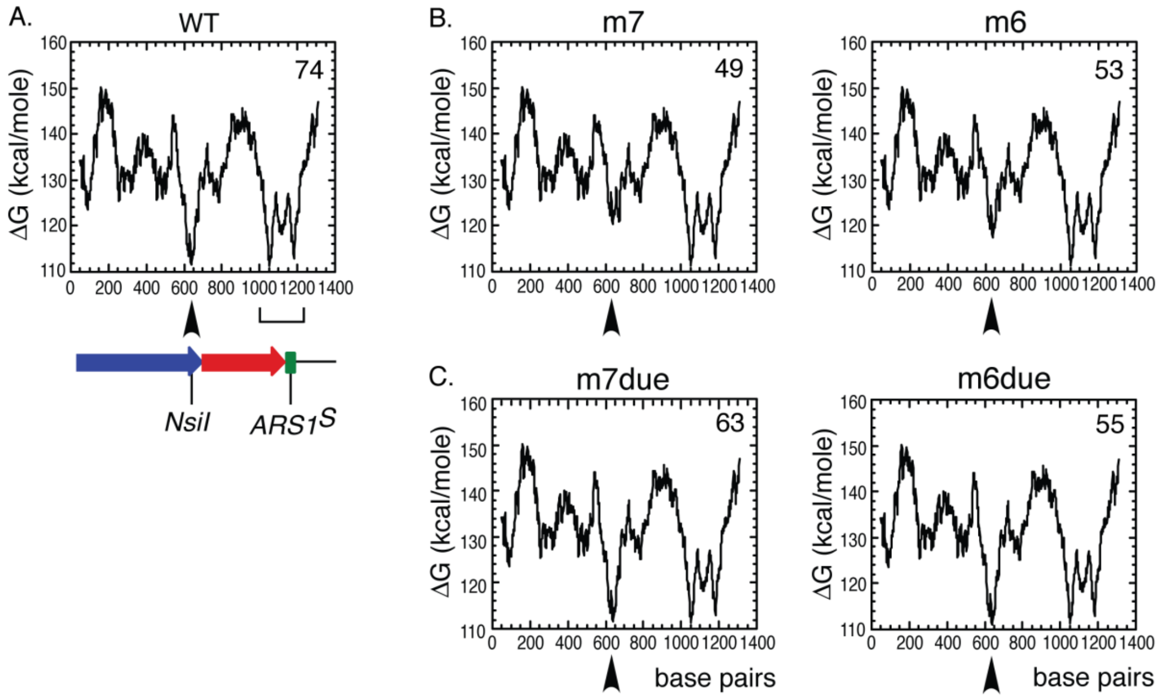

Figure S2 Examining the importance of a DUE at the bias determinant. Helical stability plots depicting the energy of unwinding (ΔG) of duplex DNA for the region containing the bias determinant (black arrow head). The percent of *ARS1*^{S} usage is depicted in the upper right corner. The cartoon depicts the sequence that was analyzed: the 3' end of *URA3* (blue arrow), the 3' end of *TRP1* (red arrow) and *ARS1*^{S} (green box). (A) Helical stability for the WT bias determinant and its surrounding region. The bracket indicates the *ARS1* DUE. (B) Helical stability for bias determinant mutants m6 and m7. (C) Helical stability for bias determinant mutants (see Materials and Methods) that maintain the presence of DUE but lack *ARS1*^{S} bias.
